# Acute and Long-Term Physiological and Hematological Responses in Well-Trained Young Swimmers Throughout a Training Season

**DOI:** 10.3390/life16030413

**Published:** 2026-03-03

**Authors:** Dimitra Nafpaktitou, Anastassios Philippou, Nikos Vagiakakos, George Vagiakakos, Alexandros Nikolopoulos, Markos Mantaloufas, George Chrousos, Michael Koutsilieris, Theodoros Platanou

**Affiliations:** 1 School of Physical Education and Sport Science, National and Kapodistrian University of Athens, 17237 Athens, Greece; nafpaktitoud@cosmotemail.gr (D.N.); al.nikolopoulos@gmail.com (A.N.); mantaloufas@gmail.com (M.M.); tplatan@phed.uoa.gr (T.P.); 2 Department of Physiology, Medical School, National and Kapodistrian University of Athens, 11527 Athens, Greece; mkoutsil@med.uoa.gr; 3 Department of Physics, National and Kapodistrian University of Athens, 15784 Athens, Greece; nvagiakakos@gmail.com; 4 School of Civil Engineering, National Technical University of Athens, 10682 Athens, Greece; gvagiakakos@gmail.com; 5 Hellenic Swimming Federation, 17672 Athens, Greece; 6 University Research Institute of Maternal and Child Health & Precision Medicine, Medical School, National and Kapodistrian University of Athens, 11527 Athens, Greece; chrousos@gmail.com; 7 Clinical, Translational and Experimental Surgery Research Centre, Biomedical Research Foundation Academy of Athens, 11527 Athens, Greece

**Keywords:** acute and chronic effects, exercise-induced adaptations, hematological parameters, physiological parameters, swimming training, young athletes

## Abstract

The physiological and hematological responses to exercise and the corresponding adaptations in high-level sports have become an important issue, from both the health and the physical performance points of view. This study investigated the fluctuations in physiological and hematological variables of young swimmers throughout a training season. Twelve well-trained male swimmers (age: 14 ± 0.3 y) participated in the study. Measurements were carried out at the beginning of the training season (T1) and pre and post the taper of each of the two competitive periods (i.e., T2, T3 for the first training macrocycle, and T4, T5 for the second macrocycle, respectively). At each of the above time points, maximum oxygen uptake (VO_2max_) was estimated, and blood samples were collected before and 1 h post a maximal 400 m swimming testing to measure hemoglobin (Hb), hematocrit (Hct), mean corpuscular volume (MCV), mean cell hemoglobin (MCH), mean cell hemoglobin corpuscular (MCHC), platelets (PLT), red blood cells (RBCs), and albumin (Alb). Adjustment for exercise-induced plasma volume changes was performed before all data analyses. Analysis of variance (ANOVA) with repeated measures followed by Bonferroni post hoc analyses was used for statistics. Multiple correlations with Bonferroni correction were also performed. Significant improvement of performance from T1 to the end of the study was recorded. Moreover, significant changes in lactate concentration ([La^−^]) with significant decrease at T3 and increase at T4 were also observed. Significant interaction (pre–post-test × test condition) for Hct, Hb, MCV, MCH, and RBCs; the main effect of test condition for Hct, MCV, MCHC, PLT, and Alb; and pre–post-test for Hct, Hb, MCV, MCHC, and RBCs were observed. No significant changes for VO_2max_ and HR were recorded (*p* > 0.05). Significant correlations between MCV and MCH at T1, T2, T4, and Hct and Hb at T1, T4, T5 were found. These results indicate that swimming training throughout a season induces both acute and long-term effects on the physiological and hematological profile of young swimmers. These findings provide fundamental information about the effects of the training volume and intensity on physical performance and might be utilized as a useful source for future studies to further characterize the systemic and performance signature of training-induced adaptations during a competitive season in swimmers.

## 1. Introduction

Endurance performance depends on oxygen transport to muscle and muscular oxygen consumption. In elite endurance athletes, evidence indicates that while muscular oxidative capacity is well adapted, oxygen transport is the main limiting factor for performance [1]. Hemoglobin and red blood cell mass play a major role in the oxygen transport chain and can thereby influence endurance performance in sports depending on aerobic capacity such as swimming. Thus, hematological monitoring across a competitive swimming season could add novel insights for further characterization of the systemic and the performance signatures of the training-induced adaptations at the various training phases.

There is a consensus that competitive swimmers are classified as endurance athletes, based on studies in well-trained swimmers showing high maximum oxygen uptake (VO_2max_) and cardiac output values during both swimming and running. Moreover, skeletal muscles engaged in swimming performance have been found to exhibit high oxidative enzymes activity and to be richly supplied with capillaries [2,3,4,5]. Indeed, the swimming training mainly aims to aerobic improvement, i.e., the capacity to maintain a high percentage of VO_2 max_ for a long period of time [6] through the appropriate activation of aerobic metabolism.

Interestingly, VO_2max_, the most representative index of aerobic fitness, is considered the most important factor responsible for the inter-individual variability in endurance performance [7,8]. It could be postulated that the improvement of VO_2 max_ might reflect improvements in associated hematological parameters, such as erythrocytes number, hemoglobin (Hb), hematocrit (Hct), mean corpuscular volume (MCV), mean cell hemoglobin (MCH), mean cell hemoglobin corpuscular (MCHC), and platelets (PLT).

Specifically, red blood cells possess a variety of functions, such as the O_2_ transport from the lungs to the tissues and the buffering changes induced in blood pH by transporting CO_2_ and binding of H^+^ to hemoglobin, which may improve exercise performance. Moreover, red blood cells seem to be able to decrease peripheral vascular resistance by releasing the vasodilators nitric oxide (NO) and ATP, with the latter stimulating endothelial NO formation and causing arteriolar vasodilation and thereby augmenting local blood flow [9,10,11]. In addition, changes in hematocrit affect blood viscosity, a rise of which increases the workload of the heart, while a decrease in blood viscosity facilitates blood flow and oxygen supply to the working muscles [12,13,14].

Albumin is a “carrier” protein for free fatty acids, an important metabolic substrate for energy production during exercise. The role of plasma albumin is crucial in the maintenance of blood volume due to its abundance in plasma proteins, high colloid osmotic pressure, and low molecular mass [15,16].

To our knowledge, there are only a few studies that have performed a long-term VO_2max_ assessment over a swimming training season, and it has been recorded that the oxygen uptake can change in response to the manipulation of training stimulus, i.e., the combination of training volume, intensity, duration, and frequency [17,18], or remain unchanged with training [19,20]. Moreover, there is extensive evidence that both acute endurance exercise and training, and specifically swimming, influence the red blood cell system, inducing morphological alterations in erythrocytes [21,22,23,24,25,26,27,28,29]. Nevertheless, less is known regarding the VO_2max_ changes in association with the fluctuations in physiological and hematological variables in young swimmers throughout a training season, which is the focus of the present study.

Swimmers are involved in training and competition at very young ages and need to cope with very demanding competitive seasons. Moreover, although increases in training load can induce positive adaptations to athletes, it may, however, also lead to maladaptation and overtraining. As physiological and hematological parameters are indices for physical conditioning and crucial for physical performance [30], and have been scarcely examined in swimming, it is essential to examine how such variables change throughout an entire swimming season. Thus, the aim of the present study was to investigate changes in physiological and hematological parameters over the training season; we hypothesized that these changes were expected due to the implementation of different training programs, in terms of intensity and volume, during the various phases of the training periodization and due to participation in competitions. Moreover, the resting hematological status, i.e., before the maximal exercise bout, at the various time points throughout the training season was expected to provide insight into the long-term training-induced hematological adaptations over time. In addition, the pre–post maximal test responses could potentially reveal a differential acute effect of maximal exercise at the various phases of the training season due to their different training stimuli.

## 2. Materials and Methods

### 2.1. Study Design

All experimental procedures were conducted in accordance with the Declaration of Helsinki for human studies and approved by the Institutional Ethics Review Board (reference number: 751/10 November 2020). Throughout the study, the participants followed a realistic swimming training program set by the team coach, which was based on the training periodization and was not influenced by the experimental design of the study. The researchers were not interacting with the team coach throughout the study and did not interfere with the training program, as the testing schedule occurred at least with a 24 h time gap before or after a training session. The 46-week training season consisted of two competitive periods, with each one concluded with a major competition. According to the periodization model, the swimming training program consisted of repetitive phases of normal training, high training load, overload training and recovery. For the purpose of examining the acute and chronic training effects on swimmers’ physiological and hematological profile, five specific testing time points of the training season were selected, corresponding to periods of distinct changes in the training workload, i.e., at the beginning of the training season, after a 2-week transition period, for establishing the baseline values (T1) and pre- and post- tapering of each of the two competitive periods (i.e., T2, T3 for the first competitive period, and T4, T5 for the second competitive period, respectively). The phase before the taper was characterized by a gradual increase in training workload (volume, intensity, frequency), which led up to the peak of the training load (i.e., the time points T2 and T4 for each competitive period, respectively), always following a periodization approach. The taper phase was characterized by a progressive decrease in volume and high intensity training. The characteristics of the training load (volume and intensity) in each of the different phases (T1, T2, T3, T4, T5) of the training season are given in Section 3.2.

### 2.2. Participants and Setting

Twelve well-trained young male swimmers volunteered to participate in the current study, after a written informed consent had been obtained from their parents. Inclusion criteria comprised that the swimmers were non-anemic and non-iron deficient. In addition, in order to avoid variability in training regime, all participants should be coming only from the same club and be monitored by the same coach. Moreover, the participants should be characterized by sufficient regular and competitive training experience, as well as being almost of the same age, to avoid interference with biological maturation. Participation was excluded to those having an unsatisfactory medical history, taking any medication, or suffering from musculoskeletal injuries and respiratory infections for 4 weeks prior to and during the study period. All tests took place at the same time of the day (to avoid any influence of circadian variations), in a 25 m indoor swimming pool under standard water and ambient conditions (water temperature: 26 ± 0.1 °C, ambient temperature: 28 ± 0.1 °C, relative humidity: 55%). Participants were asked to keep the same nutrition pattern during the 2 days prior to test, which was confirmed by the nutritional record kept.

### 2.3. Measures

Upon arrival to the laboratory at each of the above time points, body height and weight were also measured via a stadiometer and a balance beam scale, respectively (Bilance SALUS, Milano, Italy). In addition, participants’ percentage body fat was estimated based on the skinfold thickness method described by Durnin and Rahaman [31] and applying the formulae of Siri [32]. Body mass index, lean body mass and lean mass index were also calculated.

The test for determination of VO_2max_ consisted of a maximal 400 m freestyle [18,33] timed by qualified timekeepers (Seiko, 5141, Tokyo, Japan). Heart rate was monitored and recorded continuously using a short-range telemetry sensor (Polar S610i, Polar Electro Oy, Kempele, Finland). The swimmers were instructed to take a breath approximately one stroke before the finish of the 400 m swim and to exhale breath into the breathing mask as soon as it was sealed over the face by the operator immediately after finishing. Metabolic values of VO_2_ were obtained by a portable open-circuit unit (VO_2000_ Breeze Lite, MedGraphics Corp., St. Paul, MN, USA) during the first 20 s of recovery. Gas sensors and a ventilatory-flow transducer were calibrated using gases of known concentrations before each experimental swim, following the procedure indicated by the manufacturer and the backward extrapolation (BE) method, first used by di Prampero et al. [34] in speed skating and applied to swimming by Montpetit et al. [35] was used to estimate VO_2max_. The use of the BE technique during swimming has been shown to be valid and reliable (r = 0.92), allowing the swimmer to use his specifically trained musculature fully. It is worth mentioning that the direct gas exchange method affects the mechanics of swimming, since the head of the subject remains straight, the breathing pattern is altered, and the overall technique is modified and prevents the swimmer from fully exploiting his swimming ability and technique. After the completion of the 400 m maximal test, a finger-tip blood sample was taken immediately after the swim and at the 1st, 3rd, 5th, and 7th minute of the recovery period ([La^−^]_peak_) for the determination of blood lactate concentration (Lactate Pro 2, Arkray Inc., KDK, Kyoto, Japan). Moreover, blood samples were withdrawn from an antecubital vein using a sterile technique pre- (after 30 min of seated rest) and post-testing (after 60 min of seated rest). Prior to testing, standardized warm-up procedures were performed.

The swimmers’ training schedule included 6–8 sessions/wk with a pool training volume of 16–57 km/wk and 5 h/wk of dryland training (flexibility, resistance, and swimming-specific exercises). During the 46-week swimming training season, the training content was recorded daily. Training volume and intensity were quantified as proposed by Mujika et al. [36,37] and Morgado et al. [38]. The estimated percent contribution of the different energy systems to the swimming distance covered during each of the different phases of the training season is indicated in Table 1. The training intensity was individually classified in five intensity levels, based on the speed–lactate curve derived from an incremental test to exhaustion, which was repeated throughout the swimming season, allowing training zones to reflect seasonal changes in physiological status and thus enhancing the physiological validity of zone determination. This method is widely used for adolescent swimmers. Energy system contribution was estimated indirectly from the distance accumulated in each intensity zone. Zone 1 was considered predominantly aerobic, Zone 2 as mixed aerobic–anaerobic, and Zone 3 as primarily anaerobic with a substantial aerobic contribution. These values represent theoretical estimates based on intensity-domain classification, not direct measurements of metabolic energy expenditure.

### 2.4. Blood Sampling

Blood samples were collected at the selected time points (T1, T2, T3, T4, and T5, see above) of the training season into anticoagulant K2-EDTA-coated vacutainer tubes (BD Vacutainer, BD Life Sciences-Preanalytical Systems, Franklin Lakes, NJ, USA) to analyze hematological variables. Blood collection was performed 1 h post-test (after seated rest). The hematological parameters were analyzed in automated equipment (Coulter T890; Coulter, Hialeah, FL, USA) approximately 2 h after blood drawing. All samples were analyzed simultaneously, in duplicate, the results averaged, and the mean was used for statistical analysis. Potential changes in plasma volume and whole blood biomarkers (ΔTBM) were corrected accordingly using the formulas: %ΔPV = {(Hb_pre_/Hb_post_)[(1 − Hct_post_)/(1 − Hct_pre_)]} × 100% − 100% [39] and ΔTBM = (BMpost/BMpre) × (Hbpre/Hbpost) − 1 [40], respectively.

### 2.5. Statistical Analyses

Data were first analyzed for normality using the Shapiro–Wilk test. When the normal distribution was met, ANOVA with repeated measures followed by Bonferroni post hoc analyses was used for statistics (SPSS version 15.0, Chicago, IL, USA). Sphericity was verified by means of the Mauchley test. If the assumption of sphericity was not met, the significance of the F-ratios was adjusted according to the Greenhouse–Geisser procedure when the mean epsilon correlation factor was <0.75, or according to the Huyn–Feld procedure when the epsilon correction factor was >0.75. One-way and two-by-five-way ANOVA with repeated measures followed by Bonferroni post hoc analysis were used for statistics (SPSS version 15.0, Chicago, IL, USA). In the case that sphericity was violated, the Greenhouse–Geisser correction was applied when the mean epsilon was lower than 0.75. Otherwise, the Hyum–Feld correction was used. Paired Student’s *t*-tests were used for simple main-effect analysis. When the normal distribution was not met, Friedman ANOVA was used and, in this case, post hoc comparisons were made using the Wilcoxon matched pair test. Multiple correlations with Bonferroni correction were also performed. Data are presented as mean ± standard error of the mean (SEM). The level of statistical significance was set at *p* < 0.05.

## 3. Results

### 3.1. Participants’ Somatometric Characteristics

Participants had an average regular training experience of 7 years and competitive experience of 5 years, while their detailed somatometric characteristics are presented in Table 2. These characteristics changed over the training season; height increased significantly throughout the season, while body mass, LBM, and LMI showed an increase at T5 compared to T1. Moreover, there was a significant increase in LBM from T4 to T5. The participants’ somatometric characteristics are presented in Table 2.

### 3.2. Seasonal Training Load

The characteristics of the training load (volume and intensity) are presented in Table 3.

**Figure 1:**
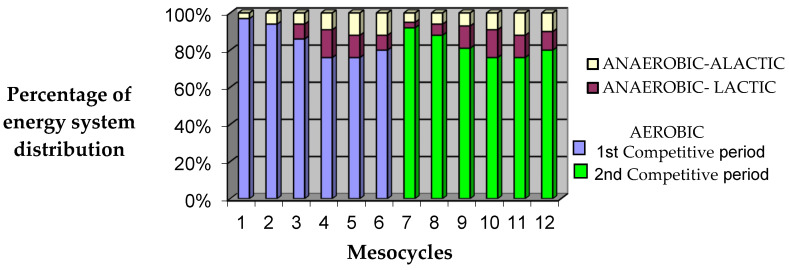
Percentage of energy system distribution throughout the training season.

The estimated percent contribution of the different energy systems to the swimming distance covered throughout the training season is indicated in Figure 1.

### 3.3. Physiological Responses

Participants’ physiological responses changed over the training season; performance time decreased significantly throughout the season, while [La^−^]_peak_ showed a significant decrease at T3 compared to T2 and a significant increase at T4 compared to T3. Moreover, there was a significant increase in VO_2max_ from T4 to T5. The participants’ physiological responses are presented in Table 4.

### 3.4. Hematological Responses

#### 3.4.1. Hematocrit

**Figure 2:**
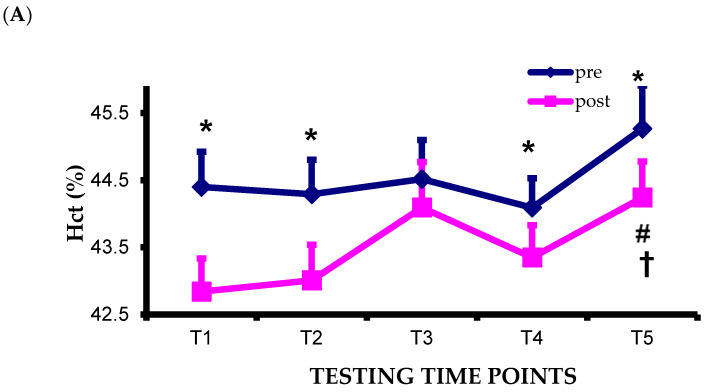

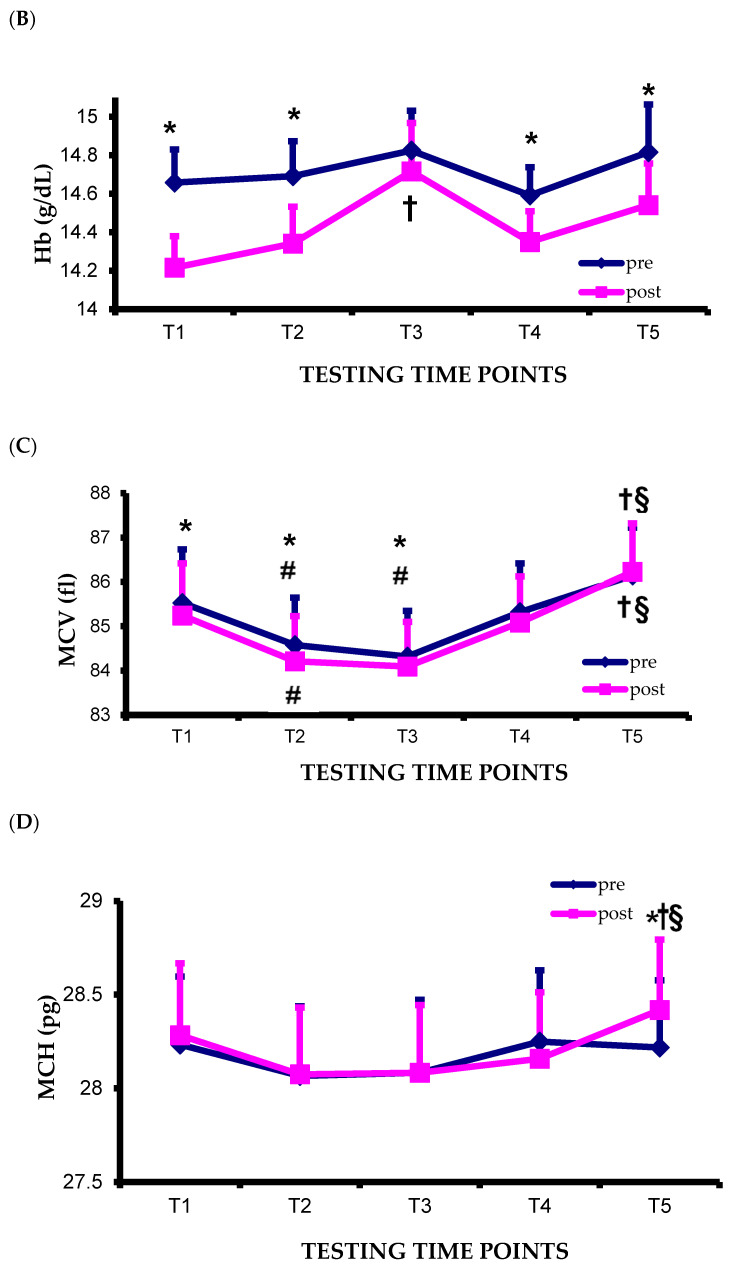

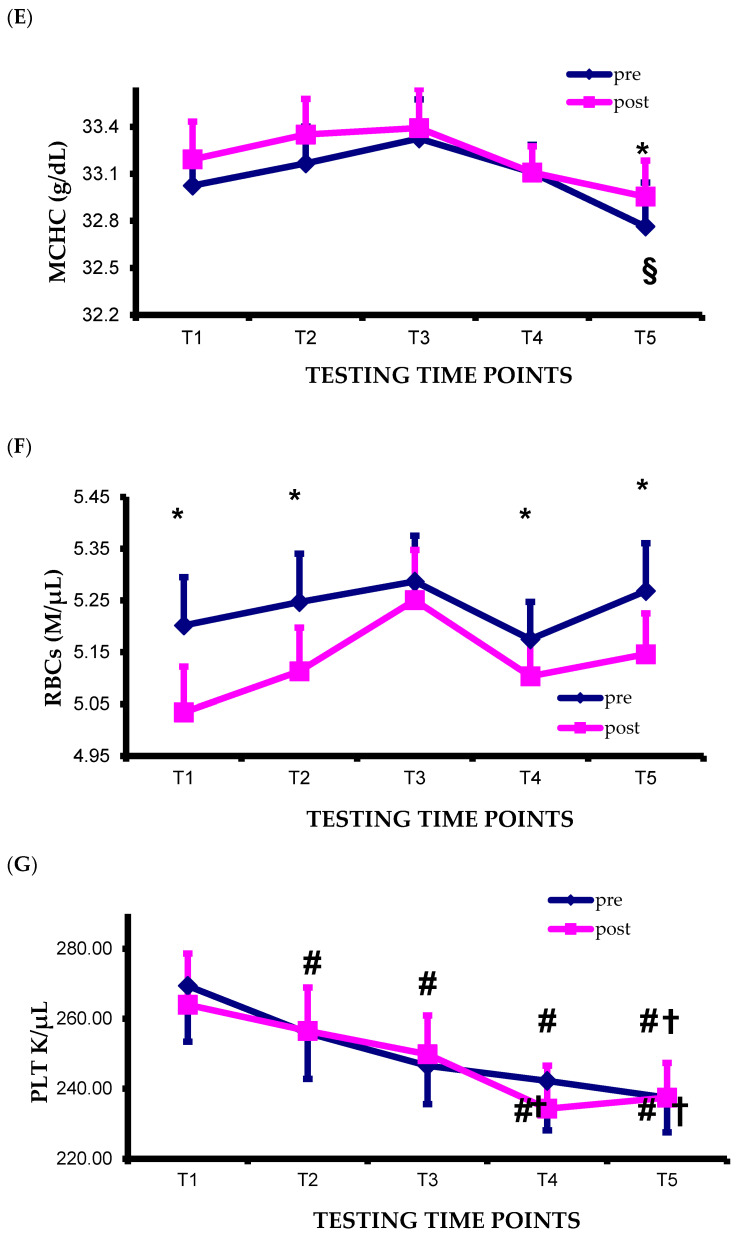

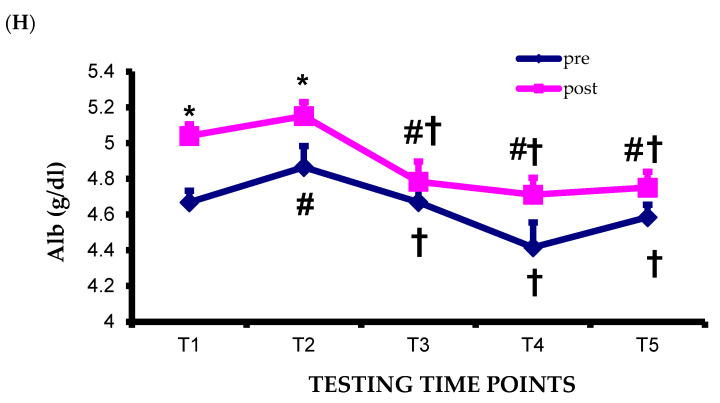
The effect of a swimming training season on (**A**) Hct, (**B**) Hb, (**C**) MCV, (**D**) MCH, (**E**) MCHC, (**F**) RBCs, (**G**) PLT, and (**H**) Alb pre- and post-400 m swim at the 5 time points. Significantly different: from pre-test at the same time point (* *p* < 0.05), from T1 (^#^ *p* < 0.05), from T2 (^†^ *p* < 0.05), from T3 (^§^ *p* < 0.05).

**Figure 3:**
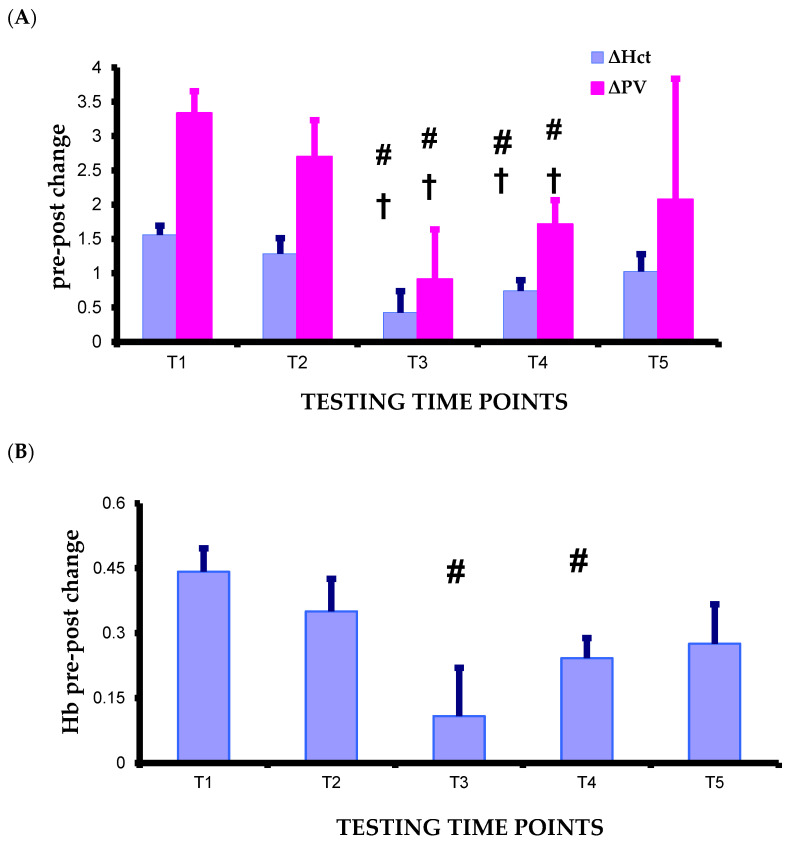

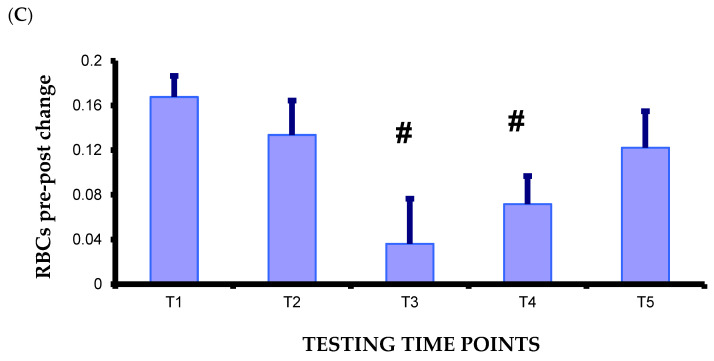
The effect of a swimming training season on pre-post-400 m swim change (**A**) Hct, PV, (**B**) Hb, (**C**) RBCs at the 5 time points. Significantly different: from T1 (^#^ *p* < 0.05), from T2 (^†^ *p* < 0.05).

Significant main effects of test condition and pre–post-test were found as well as a significant interaction between pre–post-test and test condition (F = 3.242 *p* = 0.020, F = 68.659 *p* = 0.000, F = 4.266 *p* = 0.005, respectively). Specifically, a significant post-test condition over time was found (F = 4.296 *p* = 0.005) with significant differences between the time points T1–T5, and T2–T5 (*p* = 0.040, *p* = 0.039, respectively). Significant differences between pre- and post-test at T1, T2, T4, and T5 (*p* = 0.000, *p* = 0.000, *p* = 0.001, *p* = 0.002, respectively) were also recorded (Figure 2A). Significant Hct pre–post change over time was found (F = 4.266 *p* = 0.005) with significant differences between the time points T1–T3, T1–T4, T2–T3, and T2–T4 (*p* = 0.008, *p* = 0.002, *p* = 0.038, *p* = 0.027, respectively) (Figure 3A).

#### 3.4.2. Hemoglobin

A significant main effect of pre–post-test and a significant interaction between pre–post-test and test condition were found (F = 42.890 *p* = 0.000 η^2^_p_ = 0.796, F = 2.777 *p* = 0.038 η^2^_p_ = 0.202, respectively). Specifically, a significant post-test condition over time was found (F = 3.132 *p* = 0.024 η^2^_p_ = 0.222) with significant difference between the time points T1–T3 *p* = 0.027. Significant differences between pre- and post-test at T1, T2, T4, and T5 (*p* = 0.000, *p* = 0.001, *p* = 0.000, *p* = 0.012, respectively) were also recorded. (Figure 2B). Significant Hb pre–post change over time was found (F = 2.777 *p* = 0.038 η^2^_p_ = 0.202) with significant differences between the time points T1–T3 and T1–T4 (*p* = 0.021, *p* = 0.018, respectively) (Figure 3B).

#### 3.4.3. Plasma Volume Change

A significant plasma volume (PV) change over time was found (F = 3.598 *p* = 0.013 η^2^_p_ = 0.246) with significant difference between the time points T1–T3, T1–T4 (*p* = 0.010, *p* = 0.006, respectively) (Figure 3A).

#### 3.4.4. Mean Corpuscular Volume

Significant main effects of test condition and pre–post-test were found as well as a significant interaction between pre–post test and test condition (F = 12.046 *p* = 0.000, F = 11.482 *p* = 0.006, F = 2.630 *p* = 0.047, respectively). Specifically, a significant pre-test condition over time was found (F = 10.464 *p* = 0.000) with significant differences between the time points T1–T2, T1–T3, T2–T5, T3–T5 (*p* = 0.012, *p* = 0.029, *p* = 0.015, *p* = 0.002, respectively). A significant post-test condition over time was found (F = 12.557 *p* = 0.000) with significant differences between the time points T1–T2, T2–T5, T3–T5 (*p* = 0.002, *p* = 0.005, *p* = 0.000, respectively). Significant differences between pre- and post-test at T1, T2, and T3 (*p* = 0.037, *p* = 0.003, *p* = 0.010, respectively) were also recorded (Figure 2C).

#### 3.4.5. Mean Cell Hemoglobin

A significant interaction between pre–post test and test condition was found (F = 2.862 *p* = 0.034). A significant post-test condition over time was found (F = 3.206 *p* = 0.021) with significant differences between the time points T2–T5 and T3–T5 (*p* = 0.022, *p* = 0.029, respectively). A significant difference between pre- and post-test at T5 *p*= 0.012) was also recorded (Figure 2D).

#### 3.4.6. Mean Cell Hemoglobin Corpuscular

Significant main effects of test condition and pre–post-test were found (F = 2.578 *p* = 0.050, F = 32.231 *p* = 0.000, respectively). Specifically, a significant pre-test condition over time was found (F = 2.615 *p* = 0.048) with significant difference between the time points T3–T5 (*p* = 0.031). A significant difference between pre- and post-test at T5 (*p* = 0.044) was also recorded (Figure 2E).

#### 3.4.7. Red Blood Cells

A significant main effect of pre–post-test and interaction (test condition x pre–post-test) was observed for the red blood cells (F = 37.818 *p* = 0.000, F = 3.344 *p* = 0.018, respectively). Significant differences between pre- and post-test were recorded at T1, T2, T4, T5 (*p* = 0.000, *p* = 0.001, *p* = 0.017, *p* = 0.004, respectively; Figure 2F). A significant red blood cells change over time was found (F =2.783 *p* = 0.038 η^2^_p_ = 0.202) with significant difference between the time points T1–T3 and T1–T4 (*p* = 0.017, *p* = 0.011, respectively) (Figure 3C).

#### 3.4.8. Platelets

A significant main effect of test condition was found (F = 4.333 *p* = 0.005). Specifically, a significant pre-test condition over time (F = 3.990 *p* = 0.008) with significant differences between the time points T1–T2, T1–T3, T1–T4, T1–T5, T2–T5 (*p* = 0.021, *p* = 0.043, *p* = 0.006, *p* = 0.014, *p* = 0.039, respectively) as well as a significant post-test condition over time (F = 4.475 *p* = 0.004) with significant differences between the time points T1–T4, T1–T5, T2–T4, T2–T5 (*p* = 0.003, *p* = 0.012, *p* = 0.012, *p* = 0.039, respectively) were found (Figure 2G).

#### 3.4.9. Albumin

Significant main effects of pre-test condition and after-test condition over time were found (Chi-Square = 13.597 *p* = 0.009, Chi-Square = 14.812 *p* = 0.005, respectively) with significant differences between the time points T1–T2, T2–T3, T2–T4, and T2–T5 (*p* = 0.041, *p* = 0.019, *p* = 0.010, *p* = 0.034, respectively) for the pre-test condition and between the time points T1–T3, T1–T4, T1–T5, T2–T3, T2–T4, and T2–T5 (*p* = 0.028, *p* = 0.013, *p* = 0.002, *p* = 0.004, *p* = 0.008, *p* = 0.010). Moreover, significant differences between pre- and post-test at T1 and T2 (*p* = 0.034, *p* = 0.014 respectively) were recorded (Figure 2H).

To investigate potential correlations between the hematological factors examined, specific correlation analysis was performed, which revealed significant correlations between MCV and MCH at T1, T2, and T4, and Hct and Hb at T1, T4, and T5 (*p* = 0.000).

## 4. Discussion

This study investigated the fluctuations in physiological and hematological variables of young swimmers throughout a training season. Their physiological and hematological profile was studied by a battery of parameters measured before and after a standardized (400 m swimming) maximum exercise bout in different training periods that coincided with changes in the training load throughout the entire training season and, thus, with potential variations in physical stress-induced responses. Moreover, the resting hematological levels (i.e., before the maximal exercise bout) at the various time points during the season were expected to provide insight into the training-induced hematological/physiological adaptations over time.

One of the main findings of this study was the significant decrease in Hct and Hb after the maximal exercise at T1, T2, T4, and T5. It is known that exercise causes transient fluid shifts within the body dependent on the balance between hydrostatic and osmotic forces, and there is evidence that swimming exercise results in significant hemoconcentration [41]. Indeed, during exercise the increase in blood pressure causes an increase in capillary pressure leading up to plasma efflux of the capillary beds. As swimming involves a great deal of active muscle mass, the effective capillary surface area for exchange becomes substantial. Moreover, the increase in the muscle’s osmotically active components may favor the fluids efflux [12,42]. This acute loss of plasma volume disturbing homeostasis induces physiological responses beginning almost immediately upon exercise cessation to restore plasma volume. It has been reported that the subsequent compensatory rise in PV during recovery usually exceeds the baseline PV value, and hence, results in hemodilution [43]. The mechanisms of exercise-induced hemodilution could be an increase in plasma protein levels, a decrease in central venous pressure, or an increase in renal fluid retention [44]. It has been reported that the Hct changes indicate alterations of blood plasma volume, provided that the cellular hemoglobin concentration is constant, and that the larger the plasma volume loss during the acute exercise, the greater the hemodilution after exercise [45]. According to this contention, the Hct post-test responses reflect, to some extent, the hemoconcentration that occurred during exercise. Considering the significant decrease in Hct pre–post change from the start of the study to the pre-tapering phase of the second competitive period (T1–T4: *p* = 0.002) and its no further change, it could be speculated that the greater hemoconcentration occurred at the beginning of the study (T1). On the contrary, the smallest fluid efflux and subsequent hemodilution may be at T3 since, throughout the study, at this time point the lowest Hct pre–post change was recorded. This may partly be ascribed to the significant reduction in lactate concentration at this specific time point. Moreover, although [La^−^] increased significantly at T4 compared to T3, the magnitude of Hct pre–post change was unaltered and significantly lower than the start of the study. It is speculated that this fact could be attributed to training-induced adaptations, such as the less hypotonic fluid loss from the vascular space, due to an increase in aldosterone concentration, and the increased sensitivity of antidiuretic hormone (ADH) to osmotic stimulation, both of which may lead to a reduction in hemoconcentration and subsequent hemodilution [43,46,47]. These training-induced adaptations may account for the significantly lower magnitude of Hct post value reduction at the end of the study (T5) compared to the start (T1) and the pre-tapering phase of the first competitive period (T2) (*p* = 0.040, *p* = 0.039, respectively). Moreover, an endurance training-induced significant reduction in the hemoconcentration after a bout of exercise has been reported [48]. The reduction in the magnitude of plasma volume change from the taper of the first competitive period to the end of the study indicates that the exercise-induced adaptations did not disturb homeostasis to the same extent after the swim test. Platelets are blood cells, the main physiological function of which is the formation of hemostatic plaques by adhering to damaged endothelium and releasing the content of their granules. Hence, they have a crucial role in the development of arterial thrombosis [49,50]. There is evidence that at rest platelet count is slightly lower in trained than in untrained individuals [51]. Moreover, exercise is associated with a transient platelet count increase due to the platelets released from the spleen, bone marrow and lungs [50]. In the present study, the platelet counts significantly decreased throughout the swimming training season both in the pre- and post-test conditions, which is in accordance with other studies [49,52]. This adaptation may constitute an exercise training-induced protective mechanism against the risk of thrombotic situations and adverse cardiovascular events [30].

As far as plasma albumin concentration is concerned, the current study’s findings agree with other studies demonstrating that acute exercise significantly raises plasma albumin levels, which is a potent mechanism of the exercise-induced hemodilution [53,54]. Indeed, plasma albumin pre–post test concentrations recorded a significant increase at the beginning (T1) and before the taper of the first competitive period (T2) (*p* = 0.003, *p* = 0.016, respectively). This increase in plasma albumin concentration within the first hour after the test is likely due to a mobilization of albumin from the interstitial to the intravascular space. There is irrefutable evidence that exercise has been associated with ionic disturbances in blood and muscle which are due, partly, to a redistribution of water and ions between body fluid compartments. Intense exercise results in a decrease in blood and plasma volume (hemoconcentration) as water moves from the plasma compartment into both the interstitial and intracellular fluid compartments of contracting muscle. The increase in the capillary pressure and the interstitial osmolality within the working muscles facilitate the plasma volume loss [55]. The subsequent hemodilution, a compensatory homeostatic mechanism, involves an increase in plasma albumin content in order to facilitate the fluid-flux into the intravascular space from other fluid pools, via the lymph return, and thus enhance the plasma volume restoration [43]. Specifically, exercise-induced changes in the internal environment, such as elevated deep tissue temperature, increased blood supply and pressure may slightly raise the resting diameter of capillary pores and enhance certain plasma protein passage [55]. The albumin’s low molecular mass may favor its escape from the vascular channels, entrance into intercellular spaces and eventual return to the blood by lymphatic circulation. It has been shown that the larger the reduction in plasma volume during exercise, the greater the subsequent hypervolaemia following exercise [45], and that the trained exhibited an exercise training-induced reduction in plasma volume decrease [56]. In the present study, the plasma volume expansion was smaller at T3, T4, and T5 compared with T1 and T2, which explains the non-significant pre–post-test values at these three time points. This fact indicates that the magnitude of homeostatic disturbance after the swim test was reduced at these specific time points probably due to a training-induced attenuation in osmolarity caused by the test and/or sensitivity of volume-regulating reflexes [57,58].

Plasma albumin, the most abundant protein circulating in humans, exclusively synthesized in the liver, is important for maintaining blood volume due to its ability to increase osmotic pressure and thus draw interstitial water into the intravascular space, as 1 g of albumin binds 18 mL of water. As a result, plasma volume expansion occurs, as an auto-restoration of lost plasma volume process, which usually “overshoots” the initial plasma volume and is retained for 24 h after exercise. This exercise-induced hypervolemia may be enhanced by elevated renal sodium and water retention during the 24 h post-exercise due to a rise in aldosterone concentration or, reciprocally, a reduction in atrial natriuretic peptide level. Moreover, an increase in the albumin transcription rate as well as a decrease in the trans-capillary escape rate of albumin occurs. If exercise is repeated for a period of time, the resting plasma volume “resets” to a higher level, reaching a plateau after one week of training [43,59]. This adaptation to endurance training [60] may explain the significant increase (*p* = 0.041) in plasma albumin concentration in pre-test condition found in this study at the T2 testing time point. The significant decrease in plasma albumin levels at T3, T4, and T5 compared to T2 (*p* = 0.019, *p* = 0.010, *p* = 0.034) might be attributed partly to an increased transcapillary escape rate of albumin and/or a synthesis-to-degradation ratio. Factors that influence the synthetic rate include nutrition state, hormone balance and systemic stress [61].

Red blood cells properties are directly affected during acute exercise by a number of factors including electrolyte changes, increased plasma osmolarity and viscosity by dehydration and acidosis, released free radicals, increased blood flow and blood pressure, and mechanical abrasion of their membranes due to the rapid circulation through narrow capillaries of contracting muscles. It is evident that a higher training intensity should amplify the magnitude of this effect. During longer training periods increased red cell production overcompensates for this destruction. Low-impact activities such as cycling and swimming have also been shown to induce significant levels of red blood intravascular destruction [62]. The large surface area-to-volume ratio gives red blood cells strong flexibility in order to deform and pass through the smallest capillaries in the microcirculation. However, during high-intensity exercise, the take up of metabolites such as lactate leads to red blood cells dehydration, shrinkage due to increased internal viscosity, and attenuation of their deformability, resulting in intravascular destruction [48,63,64]. This fact may explain the significant pre–post-test red blood cells values of the present study recorded at T1, T2, T4, and T5. The non-significant difference after the taper of the first period (T3) may be attributed to the significant reduction of [La^−^] concentration at this time point. Moreover, the reduced difference in pre–post values observed during the second competitive period may be attributed on the one hand to the reduction in plasma volume change after the swim test and on the other hand to a higher amount of young erythrocyte population in the bloodstream, as evidenced by the MCV, MCHC responses.

The significant reduction in MCV, in combination with the increasing tendency of MCHC in pre-testing condition during the first competitive period, suggests a progressive effect of the training load on the red blood cells lifespan. It is known that the MCHC rises with increasing red cell age as the MCV decreases, indicating an aging process [14]. In contrast, the second competitive period is characterized by a significant elevation in MCV concomitant with a significant decrease in MCHC, demonstrating the presence of newly formed red blood cells in the circulation replacing the senescent ones without, however, changing the RBC count. This contention is amplified by the non-significant correlation between MCV and MCH at T3 and T5, highlighting in this way the greatest percentage difference between senescent and old RBCs at these two particular time points. Thus, during the second competitive period the swimmers’ peripheral blood appears to contain, on average, a younger red blood cell population. This population is more resistant to osmotic or mechanical stress, has higher 2,3-diphosphoglycerate (2,3-DPG) due to increased metabolic activity and lower Hb-O_2_ affinity than the old red blood cells. As a result, the younger red blood cells improved O_2_ delivery to working muscles and O_2_ transport in the microcirculation through their enhanced deformability. The increased ability of red blood cells to pass through smallest capillaries in the microcirculation is undoubtedly of crucial importance in aerobic performance [11]. The significant reduction in MCV at pre–post testing conditions during the first competitive period may be attributed to the increased plasma osmolality that is known to cause water to move out of the erythrocytes because of oxidative damage to their major membrane components. This response may result in increased internal cytoplasmic viscosity and hence reduced deformability. The latter may contribute to the red blood cell senescence [65]. On the contrary, the non-significant change in MCV at pre–post testing conditions during the second competitive period may indicate a red blood cell turnover, having higher membrane fluidity and deformability, thus contributing to the decrease in viscosity at high shear stress facilitating the blood flow [11,14].

The significant rise in LBM at T5 as well as the apparent pool of young red blood cells that favor O_2_ unloading during exercise may partly explain the increasing tendency of VO_2max_ at this time point [11,66]. It has been well established that performance is the result of a multifactorial process. The increase in performance from the start to the end of the study with a concomitant non-significant increase in VO_2max_ may be ascribed to improvement of the technique which is, however, supported by the aerobic metabolism [67]. Evidence indicates that the aerobic trainability of adolescents, as in adult intervention studies, most likely depends on the initial fitness of participants and their training history [68]. Indeed, the experience of the swimmers participating in the present study was 7 years of regular and 5 years of competitive training. On the other hand, the contingent interindividual changes due to maturation may be masked by the overall record. It is known that aerobic improvements in adolescent males can be related to training as well as to maturation through mechanisms that are associated with hormonal changes. Consequently, alterations in body composition and increases in hemoglobin and muscle mass occur. The metabolic consequences of greater muscle mass recruited during exercise are potentially useful in providing enhanced functional capacity and possibly metabolic rate in the exercise performance. Moreover, the maturation of the central nervous system enables acquisition of sport-specific tasks and tends to produce better outcomes [68].

It has become apparent that lactate production reflects cellular stress and is one of the markers of stress response to physical exercise. Moreover, it has been found that lactate levels correlate well with pituitary–adrenal activation [69]. There is irrefutable evidence that plasma epinephrine concentration is a major effector of lactate production, through its modulation of muscle glycogenolysis and glycolysis [70]. Taper is characterized by gradually reduced training load in order to provide recovery time prior to competition, leading to a reduced SNS stimulation as a result of less relative stress So, it is plausible that the significant decrease of [La^−^] levels observed after the taper of the first competitive period (T3) may be in part attributed to the reduction in epinephrine secretion. This is consistent with the finding of Mujika et al. [71] and Hooper et al.’s [72] studies conducted on highly trained male swimmers. On the other hand, the lower plasma lactate values may also be due in part to the improved metabolic clearance of lactate as a result of the swimming training [73]. The increased [La^−^] concentration before the taper of the second competitive period may be attributed to the metabolic changes due to the increased training intensity.

A potential limitation of the study is the relatively small number of swimmers who participated in this study and were of the same gender and had been recruited from the same team. Although, this is common in training studies conducted in competitive-level populations, since long-term studies are demanding, requiring commitment, patience, and perseverance, which prevents participation; however, the generalization of the findings to the practice of competitive sports should be done with caution. On the other hand, the homogeneity of the sample and the monitoring by the same coach may be compensatory. Moreover, there are limitations regarding the swimmers’ hydration status and their dietary intake, which was confirmed by the nutritional records, though without a further quantitative analysis, although they were asked to keep the same pattern during the two days prior to testing. Iron is an essential element of Hb and is where most of this mineral is found. Smaller amounts are found in myoglobin, cytochromes and iron-containing enzymes. Stored iron is in the bone marrow, liver and spleen. Iron plays a key role in several cellular processes, and iron depletion can affect physical performance. There is no sufficient evidence in the literature that iron status decreases during a swimming season [74]. The non-assessed swimmers’ iron status constitutes also a limitation of this study, although the participants were not anemic and did not have iron deficiency.

Adolescent swimmers participating in high-load training while marked changes in physical growth occur may be at greater risk of microtraumatic injury than athletes at any other stage of development. Therefore, there may be a need to carefully monitor the training load at this age, which is characterized by rapid growth and development. The 400 m maximum effort has been suggested as a valid test to evaluate the aerobic power of swimmers in order to facilitate planning proper training periodization, considering the characteristics and specific needs of adolescent swimmers. The significant improvement of 400 m performance indicates the responsiveness of adolescent swimmers to training stimuli and suggests the meaningfulness of their regular evaluation by coaches and sports scientists over the training season.

## 5. Conclusions

This study investigated the fluctuations in physiological and hematological parameters of adolescent swimmers induced by a periodized training regime prescribed by the same coach. The findings of this study indicate that swimming training throughout a season induces both acute and long-term effects on the physiological and hematological profile within this specific cohort of young swimmers. These findings might be a useful resource for future studies to further characterize the systemic and performance signature of training-induced adaptations during a competitive season in swimmers. Such a characterization could contribute to a better understanding of the adaptation mechanisms that induce measurable long-term functional and hematological changes in swimmers.

## Figures and Tables

**Table 1:** The percentage contribution of the different energy systems, i.e., the aerobic, the anaerobic–alactic, and the anaerobic–lactic system, to the swimming distance covered in each of the different phases (represented by the selected time points T1, T2, T3, T4, and T5) of the training season.

Energy System Contribution	Testing Time Points				
	T1	T2	T3	T4	T5
Aerobic (%)	97	83	80	84.25	80
Anaerobic–lactic (%)	0	8.75	8	9	10
Anaerobic–alactic (%)	3	8.25	12	6.75	10

**Table 2:** Somatometric characteristics of the participants (mean ± SEM).

	T1	T2	T3	T4	T5
Age (y)	14 ± 0.3	14.75 ± 0.35	14.92 ± 0.34	15 ± 0.33	15 ± 0.20
Height (m)	1.69 ± 0.03	1.70 ± 0.03	1.71 ± 0.03 †‡	1.72 ± 0.02 †‡	1.72 ± 0.02 †‡
Body mass (kg)	60.9 ± 2.56	62.19 ± 2.69	62.34 ± 2.69	62.97 ± 2.74	63.49 ± 2.65 †
ΒΜΙ (kg/m^2^)	21.27 ± 0.57	21.71 ± 0.58	21.25 ± 0.56	21.22 ± 0.68	21.33 ± 0.62
Body fat (%)	16.93 ± 0.75	16.81 ± 0.82	17.41 ± 0.79	17.92 ± 0.88	17.22 ± 0.89
LBM (kg)	50.55 ± 2.09	51.67 ± 2.15 †	51.41 ± 2.10	51.56 ± 2.05	52.45 ± 2.04 †#
LMI	33.37 ± 1.39	33.96 ± 1.41	33.76 ± 1.37	33.95 ± 1.34	34.49 ± 1.34 †

BMI: body mass index, LBM: lean body mass, LMI: lean body mass index, †: significantly different from T1 (*p* < 0.05), ‡: significantly different from T2 (*p* < 0.05), #: significantly different from the previous time point of evaluation (*p* < 0.05).

**Table 3:** The characteristics of the training load (volume and intensity) in each of the different phases (represented by the selected time points T1, T2, T3, T4, and T5) of the training season.

Training Load	T1	T2	T3	T4	T5
Km/week	16.68 ± 4.26	42.31 ± 3.71	26.45 ± 3.37	40.49 ± 5.19	28.82 ± 1.58
AUL/week	8.97 ± 0.35	19.33 ± 1.67	15.73 ± 0.79	16.50 ± 1.71	15.89 ± 0.90

Km: kilometers; AUL: arbitrary units of load.

**Table 4:** Changes in the physiological parameters and performance (mean ± SEM) in each of the different phases (represented by the selected time points T1, T2, T3, T4, and T5) of the training season.

Physiological Parameters	T1	T2	T3	T4	T5
VO_2max_ (mL/min/kg)	57.91 ± 0.74	58.29 ± 0.73	58.32 ± 0.65	57.90 ± 0.73	58.42 ± 0.76
HR_peak_ (beats/min)	187 ± 3.09	184 ± 7.45	184 ± 2.52	190 ± 2.43	189 ± 2.58
[La^−^]_peak_ (mmol/L)	8.15 ± 0.71	9.25 ± 0.71	6.38 ±0.3 #	8.41 ± 0.67 #	7.73 ± 0.53
Performance (s)	306.84 ± 4.7	299.34 ± 3.8 †	300.77 ± 3.4 †	298.37 ±4.9 †	299.68 ±5.6 †

VO_2max_: maximum oxygen uptake, HR_peak_: heart rate peak, [La^−^]_peak_: peak lactate concentration, †: significantly different from T1 (*p* < 0.05), #: significantly different from the previous time point of evaluation (*p* < 0.05).

## Data Availability

All data included in this study are available upon request by contact with the corresponding author.
